# Vasospastic Angina and Role of Cardiac Catheterization

**DOI:** 10.7759/cureus.5588

**Published:** 2019-09-06

**Authors:** Robert Kleyman, Rajiv Goyal, Neha Patel, Jessica Joseph, Rami Akel

**Affiliations:** 1 Internal Medicine, Graduate Medical Education, Regional Medical Center Bayonet Point, Hudson, USA; 2 Cardiology, Graduate Medical Education, Regional Medical Center Bayonet Point, Hudson, USA

**Keywords:** prinzmetal, angina, st-elevation, coronary occlusion, transient ischemia, catheterization, percutaneous intervention, ventricular arrhythmias

## Abstract

Prinzmetal angina, also known as vasospastic or variant angina, is defined as an intermittent focal coronary artery spasm often associated with an atherosclerotic lesion near the site of spasm. It is caused by a focal or diffuse spasm of the smooth layer of the arterial wall of an epicardial coronary artery. Acute infarctions or malignant arrhythmias may develop during spasm-induced ischemia. Evaluation includes observation of echocardiogram (EKG) for transient ST elevations during discomfort; diagnosis is confirmed with coronary angiography using provocative testing. We describe two cases of patients who presented for non-cardiac complaints, but had episodes of vasospastic angina during their hospitalization. Both underwent cardiac catheterization with differing results, demonstrating the importance of catheterization in patients who experience vasospastic angina.

## Introduction

Vasospastic angina is an intermittent focal coronary artery spasm that is defined as having a transient or sustained reduction in the diameter by more than 50% in an arterial segment with insignificant (<25%) baseline narrowing [1]. It is caused by a focal or diffuse spasm of the smooth muscle layer of the arterial wall of an epicardial coronary artery [2,3]. Vascular smooth muscle hyper-reactivity is thought to be central to the pathogenesis of vasospastic angina [2,3]. The prevalence of vasospastic angina is higher in individuals from Japan compared to the Caucasian population [4]. Multiple vasoconstrictors have been used to provoke coronary spasm including acetylcholine, serotonin, histamine, noradrenaline, and dopamine, suggesting that a single receptor pathway cannot explain the phenomenon. Chest discomfort is similar to angina pain, described as being more severe and occurring typically at rest, most frequently during the hours of 12 a.m. to early morning with associated transient ST segment elevation. Each episode lasts on average from five to 15 minutes, but can be longer. There are many causes of vasospastic angina some of which include use of tobacco and recreational drugs including cocaine and amphetamine, guide wire balloon dilatation at the time of percutaneous coronary intervention (PCI), and chronic obstructive pulmonary disease (COPD) to name a few. An underlying atherosclerotic coronary artery disease (CAD) can play a role in vasospastic angina events. A recent cohort study reported that approximately 10% of patients with underlying atherosclerotic CAD presented with sudden cardiac death related to vasospastic angina events [5]. Diagnosis of vasospastic angina can be made using the Coronary Vasomotion Disorders International Study Group (COVADIS) guidelines, which includes nitrate-responsiveness, transient ischemic echocardiogram (EKG) changes, and coronary artery spasms [6].

## Case presentation

Case 1

**Figure 1:**
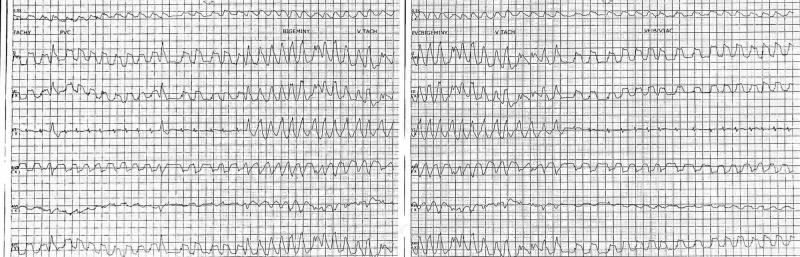
Telemetry strip demonstrating nonsustained ventricular tachycardia

A 59-year-old Caucasian male with no history of heart disease presented with shortness of breath with reported medical history of chronic obstructive pulmonary disease and tobacco use. He was treated for a COPD exacerbation without complications. On day 6 of his hospitalization, at approximately 12 a.m., he developed acute onset substernal, non-radiating chest pain with associated nausea. He denied previous history of similar symptomatology. At the time of event, he was hypertensive with a blood pressure of 190/110 mmHg, tachycardic at a rate of 150 beats per minute, tachypnic with a respiratory rate of 24 breaths per minute, saturating at 97% on three liters nasal cannula. He was actively being monitored on telemetry. At the time of symptom onset, he was found to have Torsades de pointes (figure 1) with progression to atrial fibrillation.

**Figure 2:**
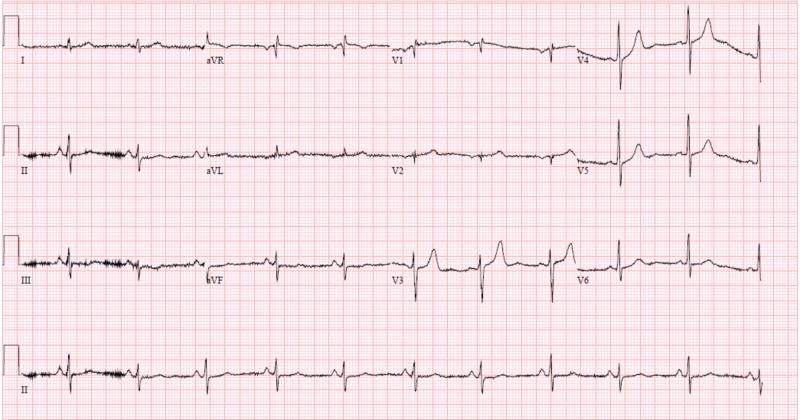
Baseline 12-lead electrocardiogram from admission

**Figure 3:**
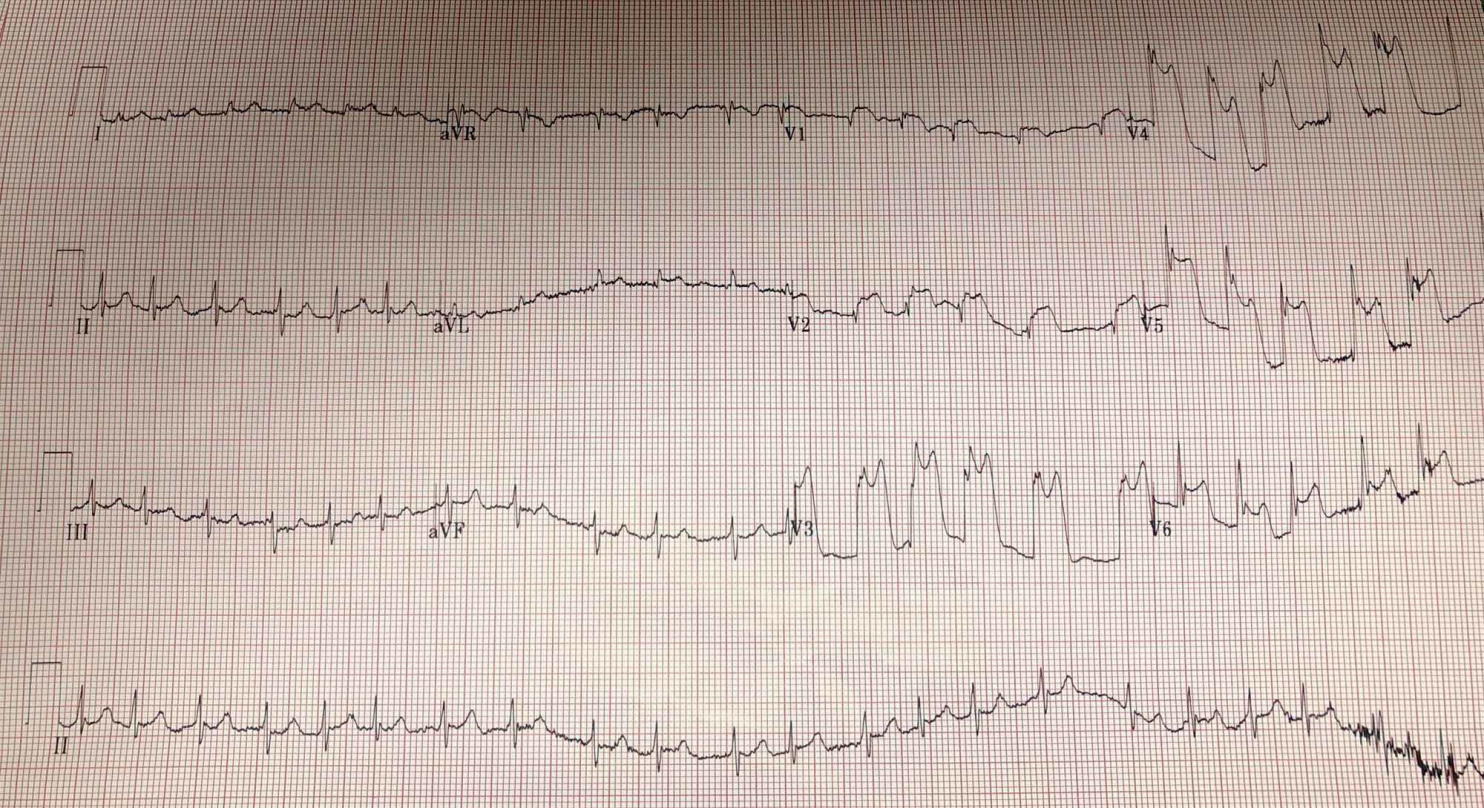
Atrial fibrillation with rapid ventricular response and significant ST-elevations in anteriolateral leads (V1-V6)

EKG obtained during the event demonstrated new onset atrial fibrillation with rapid ventricular response and significant ST elevations in the anterolateral leads that was significantly different when compared to admission EKG (figures 2-3).

**Figure 4:**
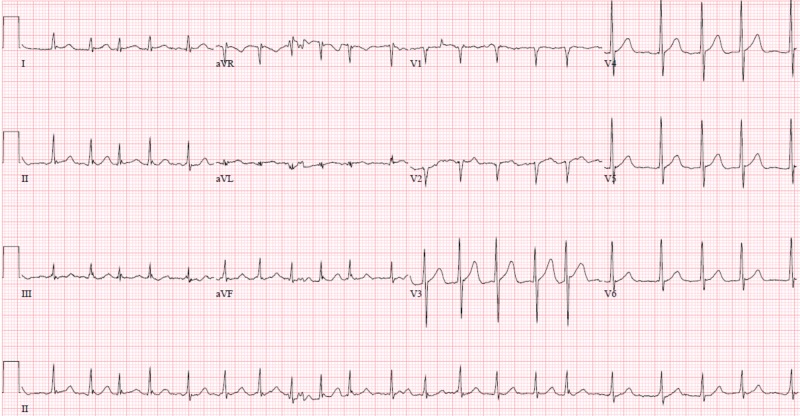
12-lead echocardiogram post nitroglycerin administration demonstrating resolution of ST-elevations

He was subsequently started on a heparin and nitroglycerin drip, along with an amiodarone drip and transferred to the intensive care unit for close hemodynamic monitoring. His symptoms resolved shortly after. A repeat EKG revealed resolution of ST elevations (figure 4).

Case 2

**Figure 5:**
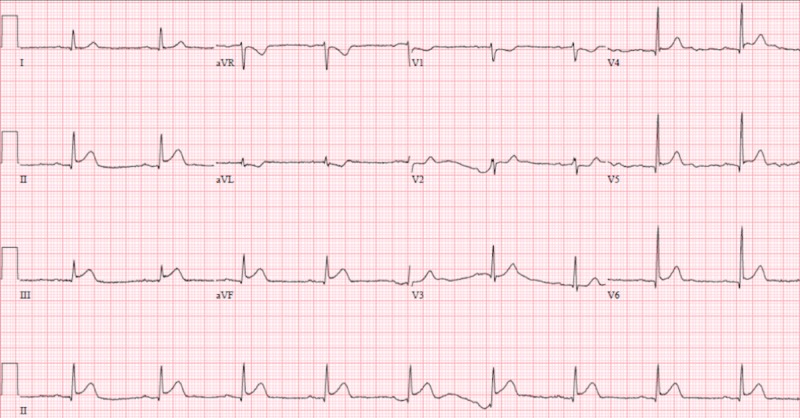
12-lead echocardiogram demonstrating ST-elevation in inferolateral leads (II, III, aVF, V5, V6)

**Figure 6:**
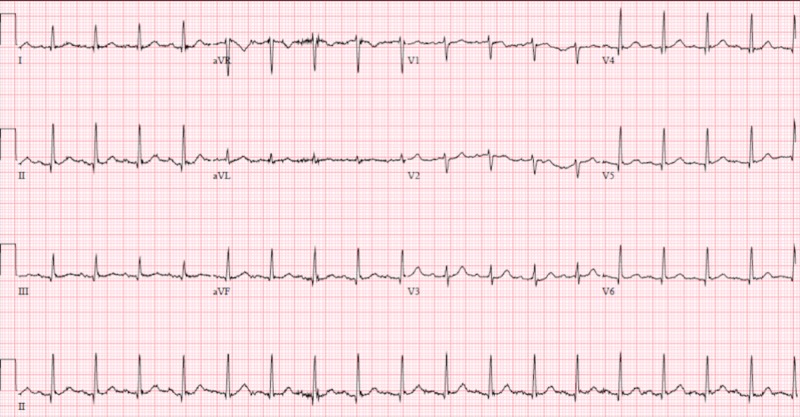
Baseline 12-lead echocardiogram from admission

A 70-year-old Caucasian male presented with lightheadedness and dizziness. He reported a past medical history of sub-massive pulmonary embolism requiring thrombolysis on anticoagulation, type 2 diabetes mellitus, recent bleeding duodenal ulcers status post argon plasma coagulation (APC) with clipping and active tobacco use. In the preceding months, he admitted to recurrent epigastric abdominal complaints described as a burning sensation that he attributed to chronic gastroesophageal reflux disease (GERD). On admission, he was found to have a hemoglobin of 4.3 g/dL and was treated for acute blood loss anemia secondary to a gastrointestinal (GI) bleed. During his hospitalization, he developed non-radiating epigastric/substernal chest pain described as a burning sensation. EKG obtained during the event revealed sinus rhythm with new ST elevations in inferolateral leads when compared to EKG obtained at the time of admission (figures 5-6).

**Figure 7:**
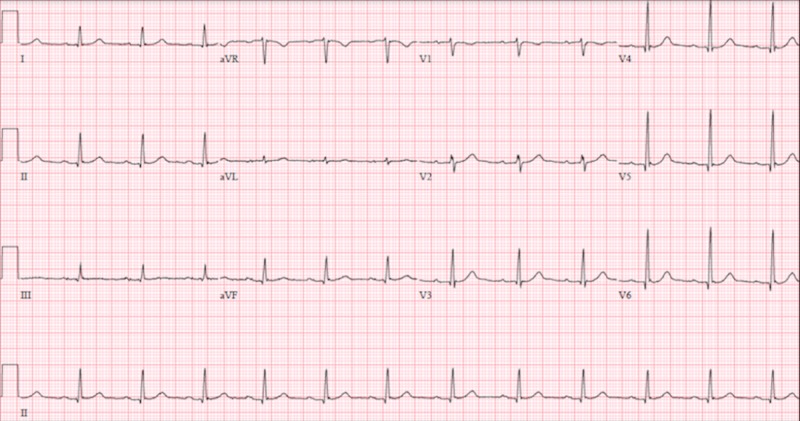
12-lead echocardiogram post nitroglycering administration with resolution of inferolateral ST-elevations

EKG findings were thought to be consistent with vasospastic angina and patient was subsequently started on isosorbide mononitrate and amlodipine. However, acute coronary syndrome could not be ruled out at the time and patient received full dose aspirin, 325 mg, and atorvastatin 80 mg. Due to GI bleed on admission, he was unable to be placed on a heparin drip. Patient’s symptoms subsided after administration of nitrate. Repeat EKG revealed resolution of ST elevations (figure 7). Troponins were obtained and peaked at 7.49 ng/mL. Patient was closely monitored for the remainder of the night and was taken for left heart catheterization in the morning.

Outcome

**Figure 8:**
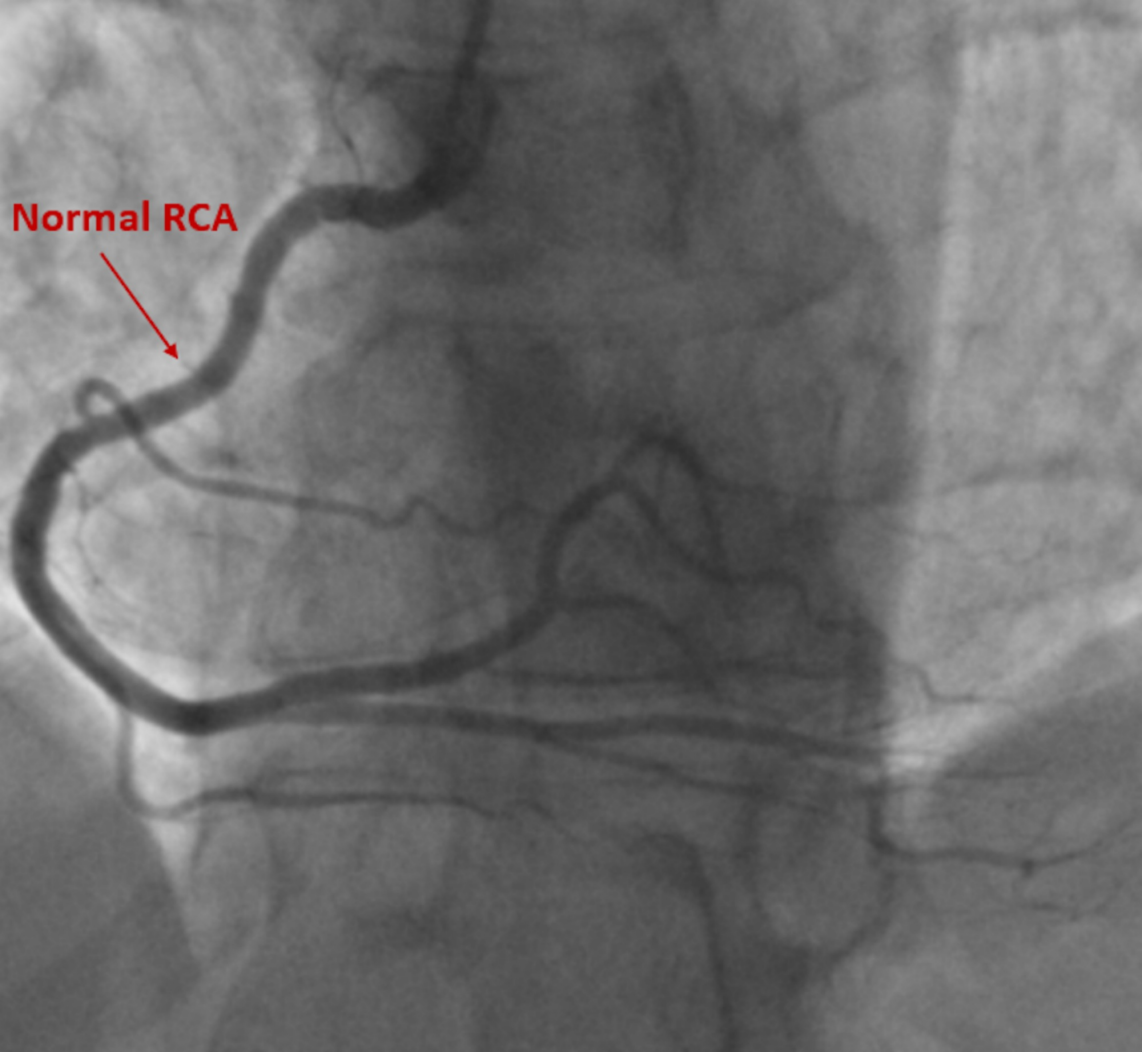
Cardiac catheterization with no evidence of disease within the right coronary artery Right Coronary Artery (RCA)

**Figure 9:**
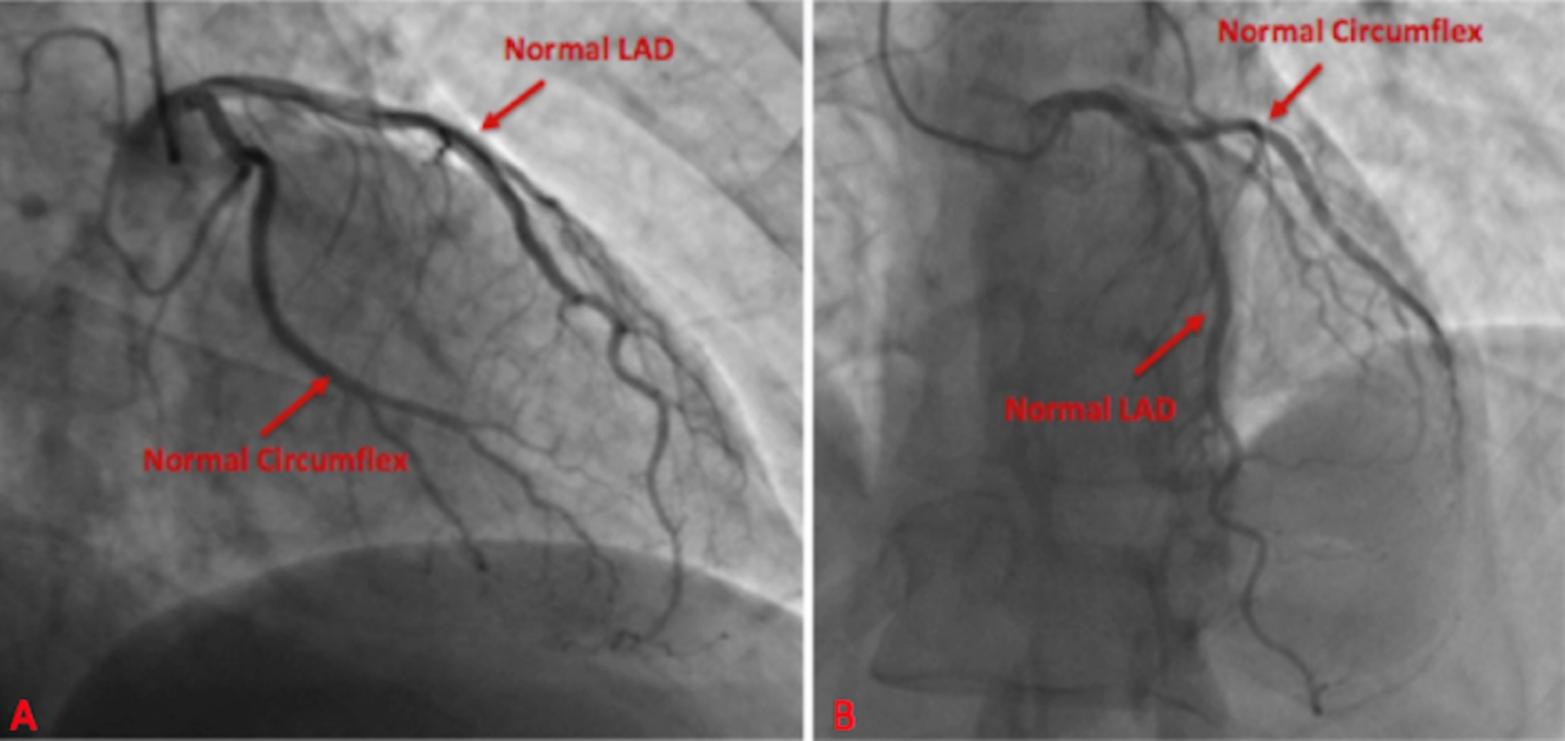
Cardiac catheterization at two viewing angles demonstrated negative disease in the left circumflex artery and left anterior descending artery Left Anterior Descending (LAD)

In each of the cases, patients were taken for cardiac catheterization. Patient in case 1 had no evidence of significant stenosis as demonstrated in figures 8-9. His vasospastic event was related to his underlying pulmonary disorder and likely active history of smoking. He was started on isosorbide mononitrate 30 mg daily, amlodipine 5 mg daily and discharged home with close outpatient follow up.

**Figure 10:**
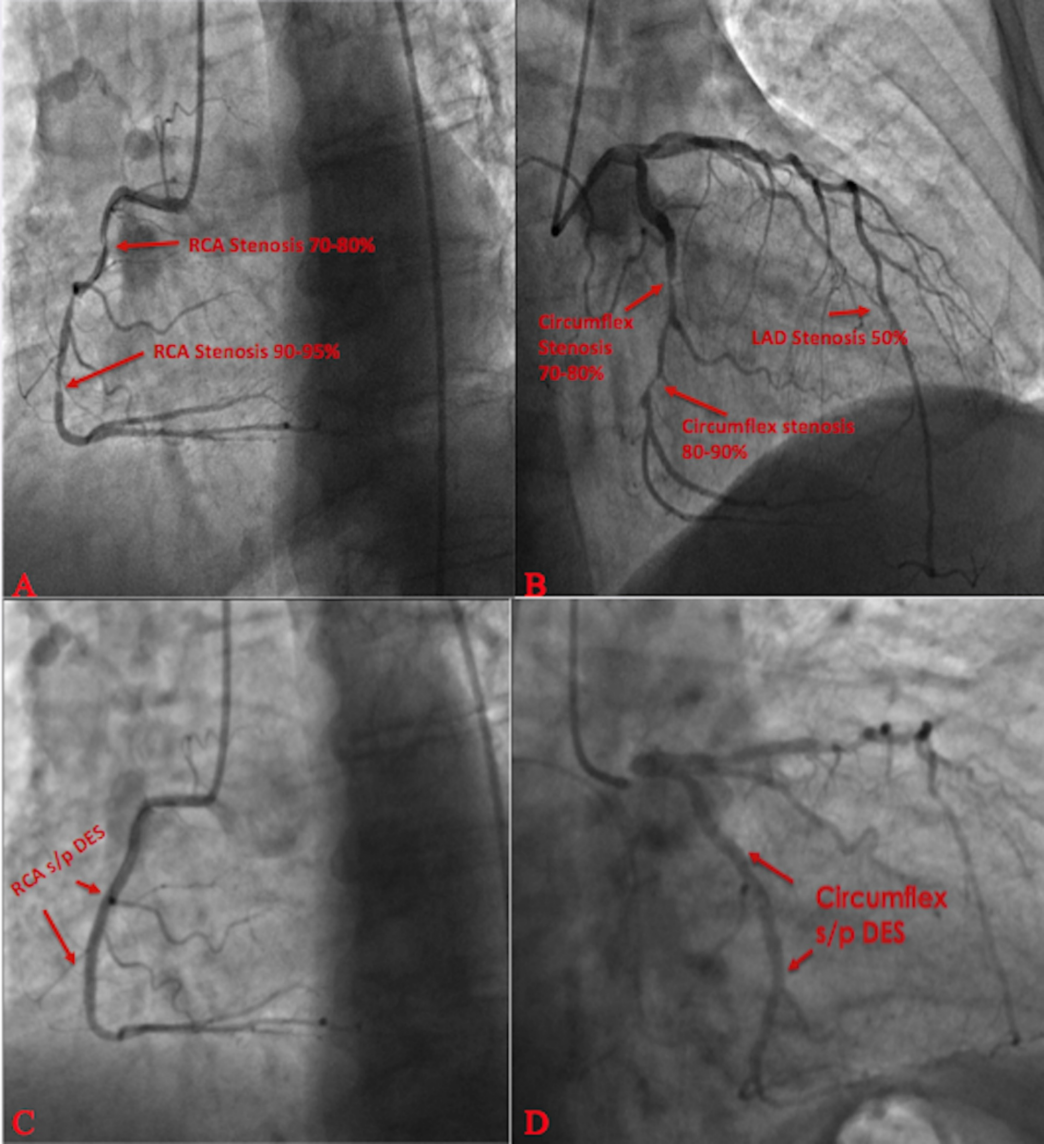
Left heart catheterization. a) Significant sequential stenotic lesions in mid right coronary artery b) Significant mid and distal left circumflex artery stenosis c) Right circumflex artery post drug eluding stent d) Left circumflex artery post drug eluding stent Status post (S/P); Drug Eluding Stent (DES); Right Circumflex Artery (RCA); Left Anterior Descending (LAD)

The patient in case 2 demonstrated multi-vessel CAD with 70-80% stenosis and 90-95% stenosis in the mid right circumflex artery (RCA) (figure 10A), along with 70-80% stenosis in mid left circumflex artery (LCX) and 80-90% stenosis in distal LCX (figure 10B). The patient successfully underwent PCI with a drug eluding stent (DES) in the RCA and a stent in the LCX (figures 10C-10D). He was started on dual antiplatelet therapy with daily aspirin 81 mg daily and clopidogrel 75 mg daily, along with atorvastatin 80 mg daily for his underlying CAD. Patient was discharged home with long-acting nitrates and calcium channel blockade for prevention and treatment of vasospastic angina events.

## Discussion

Although often a benign condition, vasospastic angina can cause malignant arrhythmias and cardiac ischemia. Causes range from tobacco use to coronary artery disease. It is important to rule out underlying CAD as the primary cause. In each case, both patients developed sudden onset substernal chest pain during their hospitalization with EKGs demonstrating ST elevations. Each patient received nitrates with resolution of their chest pain and resolution of ST elevations on subsequent EKGs, consistent with vasospastic angina. This demonstrates the importance of obtaining EKGs during the event and obtaining a repeat EKG after nitrate administration. Each patient had no previous history of CAD, but was taken for cardiac catheterization to rule out CAD as a primary cause. Patient in case 1 had no evidence of coronary disease. However, patient in case 2 was found to have severe multi-vessel disease with RCA and LCX stenosis requiring PCI with DES placement. CAD is a known cause of vasospastic angina, signifying the importance of diagnostic cardiac catheterization to rule significant cardiac disease. For patients with no evidence of significant CAD, other causes need to be excluded to prevent episodes from reoccurring. Patients who are smoking need to be counseled on importance of cessation. Regardless of underlying cause, treatment consists of long-acting nitrates and calcium channel blockers. First line treatment with calcium channel blockers and long-acting nitrates, in addition to smoking cessation, are class I indications according to American College of Cardiology [7]. Survival with medical therapy at five years can be as high as 94%, however, those with underlying CAD have an overall worse prognosis.

## Conclusions

Patients who experience an episode of vasospastic angina need to undergo further investigation in the form of a cardiac catheterization to rule out CAD as the underlying cause. It is important for clinicians to recognize vasospastic angina as this could precipitate fatal arrhythmias and cardiac ischemia resulting in increased mortality. Proper recognition, diagnosis and management can help decrease overall mortality in patients.
